# Processing of misinformation as motivational and cognitive biases

**DOI:** 10.3389/fpsyg.2024.1430953

**Published:** 2024-08-30

**Authors:** Yanmengqian Zhou, Lijiang Shen

**Affiliations:** ^1^Department of Communication, University at Buffalo, Buffalo, NY, United States; ^2^Department of Communication Arts and Sciences, Pennsylvania State University, University Park, PA, United States

**Keywords:** misinformation persistence, correction, motivational bias, cognitive fallacies, psychological inoculation

## Abstract

Misinformation can be broadly defined as false or inaccurate information created and spread with or without clear intent to cause harm. It travels fast and deep and persists despite debunking. It is well-documented that corrective messages and fact-checking efforts often fail to mitigate the effects or persistence of misinformation. In this article, we examine the persistence of misinformation as rooted in motivational and cognitive biases in information processing. While drawing on the frameworks of motivations that drive information seeking, sharing, and processing and various cognitive biases, we explicate mechanisms and processes that underlie the impact and persistence of misinformation. We conclude our article by discussing the potential utility of psychological inoculation as a prebunking strategy.

## Introduction

Misinformation, in its broadest sense, encompasses “any information that is demonstrably false or otherwise misleading, regardless of its source or intention” (van der Linden et al., 2023, p. 7). The defining characteristic of misinformation lies in its (lack of) truth value: Information that is inaccurate, incomplete, misleading, or false information as well as selective and half-truths. However, the intention remains an important dimension. Consider a white lie, information that is untrue, maybe misleading, but with a benign intention. It is rather safe to assume that white lies are not viewed as misinformation. Amid ongoing debates on the most appropriate criterion against which the true value or ground truth of a piece of information should be assessed, several key benchmarks emerge, including evidence or facts, expert opinion, and characteristics of deceptive or manipulative techniques (Nan et al., 2023; Roozenbeek et al., 2022; Vraga and Bode, 2020). While some distinguish misinformation and disinformation, with the latter being purposefully created and shared to deceive and cause harm, we consider disinformation as a subset of misinformation, recognizing the inherent challenge of detecting intent in many cases (Treen et al., 2020).

The widespread dissemination of misinformation poses one of the greatest challenges facing contemporary human society, which is exacerbated by the high-choice media and social media environment. While misinformation is not new and its history dates back to the early 15th century (Soll, 2016), its increasingly evident and severe ramifications across a wide array of public domains, including science, health, and politics, among others, have gained the phenomenon unprecedented attention in recent decades, particularly post-2016, the year when “post-truth” was selected as the word of the year by the Oxford dictionary. Indeed, misinformation has been documented to undermine electoral processes (Berlinski et al., 2023), diminish support for proclimate policies (Treen et al., 2020), fuel vaccination hesitancy (Garett and Young, 2021), and discourage the enactment of preventive and safety behaviors during the coronavirus diseases 2019 (COVID-19) pandemic (Greene and Murphy, 2021). Beyond mere facts, a pressing concern is understanding how and why misinformation influences individuals’ decision-making and yields these detrimental outcomes. Over the years, there has been a shift from a paradigm heavily emphasizing information deficits, which attributes the impact of misinformation to a lack of understanding and knowledge about facts, to a more nuanced framework recognizing that ignorance alone lacks explanatory power (Ecker et al., 2022). This transition acknowledges the mounting evidence that people persist in making decisions based on misinformation despite retractions and corrective efforts (Seifert, 2014; Thorson, 2016)—some parents’ belief in the measles, mumps, and rubella (MMR) vaccines–autism link persists long after the infamous 1998 Lancet article was retracted (in 2010) and debunked (in 2011) is a good example.

Misinformation can occur and spread without any prior position, particularly when it is due to a lack of knowledge (e.g., even though it was established in the third century by Aristarchus and Eratosthenes that the earth was round, the idea was not widely accepted until around the 15th century; Uri, 2020). It is not a coincidence, however, that misinformation is most rampant on issues that are controversial and politically charged (e.g., climate change, the 2020 election, COVID-19, etc.), given the polarization and dividedness of society in the post-2016 United States. This suggests that it is most likely that individuals already have formed their opinions and/or developed orientations toward the specific issues before misinformation is generated and spread. In this article, we take the perspective of biased and motivated information processing to understand and explain the impact and persistence of misinformation. Specifically, we examine responses to scientific evidence or corrective messages as a special case of the processing of counterattitudinal information driven by multiple motivations and cognitive fallacies. Conversely, misinformation as attitude-consistent messages undergoes similarly biased processing. It should be noted that biased and motivated processing of scientific evidence and facts by individuals with the correct positions do not have any other undesirable consequences (e.g., Shen et al., 2009), although they equally contribute to the polarization and dividedness of society as those to whom misinformation is attitude-consistent. There is evidence that greater partisan divisions in social reality (Nyhan, 2021) and a stronger desire for shared reality (Jost et al., 2018) lead to increased susceptibility to misinformation and willingness to share/spread misinformation. Hence, we have Proposition 1:

**Proposition 1**: Misinformation is more prevalent, influential, and persistent on topics/issues that are controversial and politically charged than neutral or non-divisive ones.

Admittedly, misinformation often has attention-grabbing features that make it attractive and easy to process. For example, compared to factual news, misinformation tends to be more emotional and less lexically diverse and demonstrates greater readability (Carrasco-Farré, 2022). It is often high in sensationalism (Staender et al., 2022) and contains negative or threatening content, social cues, and the presence of celebrities (Acerbi, 2019). In addition, misinformation frequently employs manipulative techniques. A systematic review identified the use of logical fallacies and misrepresentations, cherry-picking, fake experts, impossible expectations, and conspiracy theories as common message features shared by most misinformation (Schmid et al., 2023). Roozenbeek et al. (2022) outlined five epistemologically dubious strategies commonly observed in online misinformation, including (1) the use of emotionally charged language that evokes fear, anger, and other negative emotions, (2) the presentation of incoherent or mutually exclusive arguments, (3) the framing of issues in false dichotomies, (4) the scapegoating of individuals or groups to reduce the complexity of a problem, and (5) the resort to *ad hominem* attacks that target the speaker rather than their arguments. Undoubtedly, these features of misinformation are conducive to the spread and persistence of misinformation (Kemp et al., 2022; Putman et al., 2017). Yet, cognitive theories suggest that it is primarily through individuals’ cognitive processing that misinformation persists while correction fails. That is, these message features feed into the motivational and cognitive biases in information responses, especially when individuals already hold a preexisting belief in misinformation, which then shapes the outcomes of the messages. Recognizing the influence of these message features, we now turn our discussion to the motivational and cognitive biases that underpin the persistence of misinformation.

## Motivational and cognitive mechanisms

Multiple motivational and cognitive mechanisms may contribute to how people engage with misinformation and corrective information (Desai et al., 2020; Kunda, 1990). We cluster these mechanisms into three groups. The first one consists of more deliberate motivations. These motivations concern goals that individuals actively or consciously pursue to achieve a desired state. The second category includes more automatic motivations. It differs from the first one in that these goals tend to be triggered automatically and the goal pursuit might be more or less unconscious. The third class of mechanisms centers on cognitive fallacies, which refer to a type of information processing that reduces accuracy and results in erroneous and/or irrational conclusions. Cognitive fallacies are distinct from the motivations and can occur absent any motivations.

### Deliberate motivations

Humans operate on three fundamental motivations: value, control, and truth (Cornwell and Higgins, 2018). These motivations are essential to how people seek effectiveness (Higgins, 2014). Value motivation is concerned with having desired results (i.e., expected utility), especially as they relate to avoiding pain and cost and gaining pleasure and benefits. Control motivation involves making things happen and managing what happens and how it happens. Truth motivation pertains to the establishment of what is real vs. imaginary and what is right vs. wrong (Cornwell and Higgins, 2018). All three motivations suggest that individuals are *not* intrinsically motivated by accuracy as the dual process models (Chaiken et al., 1989; Petty and Cacioppo, 1986) suggest they might: Even the truth motivation involves subjective and value-laden judgments (i.e., what is right vs. wrong); although conceivably, the motivations of value and control may play a particularly crucial role in the creation, spread, and persistence of misinformation. Individuals may generate, biasedly process, and disseminate misinformation and avoid interacting with others who disagree with them to secure desired outcomes and exert control. This could be especially true among those who already hold misinformed beliefs. By perpetuating misinformation and resisting corrective efforts, they fulfill their goals of preserving a favorable status quo, avoiding unwanted threats, and potentially enhancing their influence and gaining dominance (Cornwell et al., 2014). The subjectivity in, or worse yet, the lack of, truth motivations further explains why corrective information presenting facts and evidence often fails to persuade—accuracy and validity do not necessarily satisfy the truth motivation (i.e., when they are evaluated along the dimension of right vs. wrong). Facts and evidence tend to be ignored, and their sources are rejected when they do not render the value (i.e., expected utility) or control (cf. Festinger, 1957).

In line with Higgins and colleagues’ tripartite framework of motivation, Sharot and Sunstein (2020) identified instrumental (action), hedonic (affect), and cognitive (cognition) utilities as three motivations that guide people’s informational behavior. People are motivated to acquire and accept information they perceive as facilitating decision-making and actions that maximize rewards and minimize harm, inducing positive emotions evading negative ones, and enhancing their ability to understand and predict realities. Instrumental and hedonic utilities operate in similar ways as control and value motivations: The pursuit of action-facilitative knowledge and positive affect can drive individuals to adopt misinformation and resist corrective information. Indeed, people manage what information to seek, accept, and believe in as an emotion regulation strategy (Heinström et al., 2022; Vrinten et al., 2022). Cancer patients may be motivated to endorse treatment-related misinformation due to the hope it gives. When driven by hope, people experience reduced message fatigue (Shen et al., 2022), displaying increased openness to (mis)information they consider useful. Considerations of cognitive utility—more specifically, the motivation to minimize the gap between one’s mental representations and external reality and thus have a secured sense of comprehension—may lead to avoidance of information that threatens existing mental models (Sharot and Sunstein, 2020). Consequently, corrective information tends to be assigned negative cognitive values whereas misinformation is considered to have positive cognitive values to satisfy the need to align internal cognitions with (distorted) external realities.

Despite the differences, the more recent theorizations on motivations are not incompatible with the dual process models. The heuristic and systematic processing model (HSM) propose people may be driven by accuracy, defense, and impression motivations (Chaiken et al., 1996), which are, respectively, aligned with outcome-, value-, and impression-relevant involvement (Johnson and Eagly, 1989). Accuracy motivation is the desire to arrive at valid attitudes or beliefs that correspond with reality. It is closely associated with outcome-relevant involvement where one’s attitudes are primarily concerned with direct personal consequences and concrete utility. Accuracy motivation promotes in-depth and careful processing of information that allows one to reach accurate conclusions. Defense motivation refers to the desire to hold attitudes or beliefs that are consistent with self-definition. Associated with value-relevant involvement where a person’s attitude is primarily linked to one’s self-identity and values, defense motivation drives people to process information in ways that preserve their self-definition and -concept. Impression motivation is the desire to express attitudes and beliefs that satisfy interpersonal and social goals. It corresponds with impression-relevant involvement that highlights the self-presentational and social–relational consequences of one’s expressed attitudes. When impression-motivated, people engage in processing strategies that yield conclusions that enable social acceptance. In the case of misinformation, motivations other than accuracy presumably underlie individuals’ processing. As individuals form a preexisting belief in misinformation, particularly on highly controversial or polarized issues, their position becomes closely tied to their personal values, identities, and social belongingness, which—when confronted with correction—triggers defense and/or impression motivations and thereby leads to biased processing that allows them to arrive at conclusions that favor their existing misconceptions (Jost et al., 2022; Trevors, 2019). Accuracy motivation, an assumption central to corrective messages presenting factual evidence, on the contrary, is absent or overshadowed, limiting the utility of corrective efforts. Hence, we have Proposition 2:

**Proposition 2**: The impact and persistence of misinformation are positively associated with non-accuracy motivations.

### Automatic motivations

Cognitive consistency theories, such as balance theory (Heider, 1946), congruity theory (Osgood and Tannenbaum, 1955), and cognitive dissonance theory (Festinger, 1957), share a common assumption that people are driven to maintain consistency among elements of their cognitions (i.e., units of information). The goal of restoring consistency tends to be evoked by the psychological discomfort resulting from inconsistency and may occur beneath conscious awareness without deliberate intent. Balance theory, for example, postulates that balance—a psychologically pleasant, desirable state—is achieved when all cognitive elements are internally consistent. More specifically, often studied within the P-O-X framework where P refers to the person/self, O refers to the other person, and X refers to the object, balance is described as the state in which a person (dis)agrees with another person they (dis)like on the attitudinal object (Heider, 1946). When in an imbalanced state, people are motivated to change one or more relationships in the triad. Congruity theory shares similar propositions as balance theory. However, it is more precise than balance theory in that it quantifies the degree of liking of the other person and one’s attitude toward the object (Osgood and Tannenbaum, 1955). As such, congruity theory allows a more accurate prediction.

Cognitive dissonance theory differentiates three types of relationships between cognitive elements: irrelevant, consonant, and dissonant (Festinger, 1957). As the ratio of dissonant to consonant cognitions and the importance of dissonant elements increase, individuals experience greater cognitive dissonance and greater motivation to reduce it. Correspondingly, the reduction can be achieved in ways such as adding new consonant cognitions, removing dissonant cognitions, and changing the importance of consonant and/or dissonant cognitions. These cognitive consistency theories suggest that when there is a discrepancy between the advocacies of the message and individuals’ preexisting positions, people are automatically motivated to close that gap, which likely leads to message resistance and rejection. This means that when a belief in misinformation is established, any corrective information threatens the preferred state of consistency and arouses motivations to dismiss, distort, or deny it. People may also seek and internalize information that reinforces their existing misconceptions. Within the framework of cognitive dissonance, individuals who already believe in misinformation might still be open to facts and evidence, that is, when there are external justifications that reduce cognitive dissonance. However, even when that happens, the impact of facts and evidence is most likely in the form of compliance instead of identification or internalization (Kelman, 1958). Attitude change in the form of compliance tends to be flimsy and short in duration, which explains why fact-checking might have a short-term effect in mitigating misinformation but is positively associated with the persistence of misinformation (Chan et al., 2017).

A closely related theory to cognitive consistency theories is the self-affirmation theory (Steele, 1988). Its distinguishing feature lies in its premise that people are primarily driven to maintain self-integrity (rather than cognitive consistency). Self-integrity is the perception of the self “as adaptively and morally adequate, that is, as competent, good, coherent, unitary, stable, capable of free choice, capable of controlling important outcomes, and so on” (Steele, 1988, p. 262). When encountering information that threatens one’s perceived self-integrity, people are motivated to restore it. Although accepting the information and changing one’s attitude is an option, it is often unlikely due to the threat it poses to the fundamental aspects of one’s identity. Rather, people are more inclined to engage in defensive responses, which can be automatic in nature, to avoid or dismiss the threat (Sherman and Cohen, 2006). This process clearly sheds light on why beliefs in misinformation tend to be resistant to correction. However, it is worth noting that divergent from cognitive consistency theories, self-affirmation theory proposes a third alternative adaptation mechanism, which is to self-affirm a different domain of identity that is not necessarily related to the threat (Steele, 1988). Putting this differently, when self-integrity is bolstered through self-affirming some alternative aspects of the self, people may find cognitive inconsistency tolerable and respond more openly and objectively to threatening information (Sherman and Cohen, 2006; Steele and Spencer, 1992). Nevertheless, this seemingly promising tenet may encounter challenges in practice given the polarization of society across various interconnected issues and the intricate intertwining of important domains of individuals’ identities, which often align with one another. This can undermine the applicability of this adaptation, giving way to defensive responses.

Social judgment theory (Sherif et al., 1965) is a third relevant framework in this cluster. The theory posits that attitude change is a function of one’s judgment of the position advocated by a message in relation to their existing position. Specifically, depending on where an individual’s initial position is located on a continuum of all potential alternative positions, one might evaluate a message’s advocacy as within their latitude of acceptance (i.e., positions that one considers acceptable), rejection (i.e., positions that one considers unacceptable), or non-commitment (i.e., positions that one considers neither acceptable nor unacceptable). Attitude change is likely to be greatest when a message’s advocacy is most distant from one’s existing position but does not cross into the latitude of rejection. When a position falls within the latitude rejection, conversely, persuasion is unlikely and may even cause backfire. Corrective messages as counterattitudinal information reside in the latitude of rejection, eliciting automatic resistance against the message and subsequently the observed ineffectiveness. Moreover, social judgment theory suggests that the size of the latitude varies based on the level of ego involvement. As the issue in question is of greater personal importance to an individual (i.e., greater ego involvement; Sherif et al., 1973), which is frequently the case given the highly politicized and divided nature of topics on which misinformation proliferates, the latitude of rejection expands, heightening the likelihood of contrast effect wherein an advocated position is perceived more distant from their position than it actually is (Sherif et al., 1965; Sherif and Hovland, 1961). Consequently, existing beliefs in misinformation fail to be corrected and might be further reinforced.

There has been evidence that conservatives are more likely than liberals to prioritize values of conformity and tradition, possess a stronger desire to share reality with like-minded others, and perceive within-group consensus when making judgments (Jost et al., 2018). Along with the features of misinformation such as false dichotomy, scapegoating, and hominem attacks (Roozenbeek et al., 2022), these motivational tendencies of the conservatives make them more susceptible to misinformation than liberals (Garrett and Bond, 2021), although both conservatives and liberals are gullible (e.g., van der Linden et al., 2020). Conservatives are also more likely than liberals to be influenced by relational cues and sources who are similar to themselves, maintain homogenous social networks, and favor echo chamber environments (Jost et al., 2018). Hence, we have Propositions 3 and 4:

**Proposition 3**: Consistency- and self-identity-related motivations are positively associated with misinformation effects and persistence.

**Proposition 4**: The impact of misinformation is more pronounced and its duration more persistent among conservatives than liberals.

### Cognitive fallacies

Biased responses may occur due to cognitive fallacies in the absence of motivations (MacCoun, 1998). A wide range of cognitive fallacies might be relevant to understanding the persistence of misinformation within the framework of the processing of counterattitudinal information. Here, we focus on a few most pervasive ones that are closely related to (selective) exposure to information and the subsequent biases in decision-making.

Epistemic egocentrism is a form of perspective-taking failure where individuals are unable to set aside their privileged information that they know is unavailable to others, leading to predictions that skew others’ perspectives toward one’s own (Royzman et al., 2003). This tendency means that people have a hard time adopting viewpoints that are not their own and struggle to process and understand counterattitudinal information, such as corrective information (Shen and Zhou, 2021). The failure to take others’ perspectives may closely interplay with the conviction that one’s own perspectives objectively reflect reality and that those who do not share the same perspectives are uninformed and incompetent, a bias termed objectivity illusion (Schwalbe et al., 2020). The illusion that one is immune to bias (see also biased blind spot, Pronin et al., 2002) exacerbates the difficulty of rectifying misconceptions as attitude-incongruent messages are dismissed as distorted and irrational.

Choice blindness is a fallacy closely related to epistemic egocentrism. Choice blindness refers to the inability to detect a discrepancy between one’s intended choice and the choice presented to them (Johansson et al., 2005, 2006). It is the tendency to be unaware that one’s choices and preferences have been changed or manipulated after the decision is already made. Epistemic egocentrism leads people to (1) look inward to examine their own thoughts and inner states such as emotions, preexisting judgments, and perceptions, (2) (incorrectly) believe they fully understand the roots of their inner states, but (3) assume that other people’s introspections are largely unreliable. When people have formed their positions and opinions based on misinformation, more or less due to manipulations, choice blindness means they tend to rationalize and justify this manipulated decision and adjust their attitudes to align with the misinformation (Stille et al., 2017), while deeming (counterattitudinal) facts and evidence as from other people’s inner states, hence largely unreliable.

Confirmation bias, is the tendency to seek, interpret, and remember evidence in ways that favor existing beliefs or expectations (Nickerson, 1998; Oswald and Grosjean, 2004), can be considered a special case of epistemic egocentrism. It can occur without the presence of motivations and, in some cases, involuntarily, such that people may fall for this bias even when they have no obvious personal interest (Gilead et al., 2019; Nickerson, 1998), leading misinformation-believing individuals to reject correction regardless of their involvement (Zhou and Shen, 2022). One consequence of confirmation bias is selective exposure, a tendency for individuals to preferentially seek, attend to, and engage with information that is consistent with their inner states (i.e., preexisting beliefs, values, and attitudes), positive in valence, high in sensational value, and easy to process. Often co-occurring with confirmation bias and selective exposure is another cognitive fallacy, illusory correlation. Illusory correlation describes the fallacy of perceiving a correlation where none exists or perceiving a stronger correlation than it really is (Hamilton and Rose, 1980). The (mis)belief in the MMR vaccine–autism link and the (mis)belief that the influenza vaccine gives one the flu are good examples of illusory correlation. It should be noted that illusory correlation happens not only to lay individuals but also to well-trained social scientists—type I error is not uncommon. Publication bias and self-confirmation bias often drive some scientists to cling to their theories in the face of disconfirming data that call for the rejection of their own theories (Kuhn, 1962). Underlying this bias is the process where preexisting attitudes or beliefs prime individuals to search for supportive evidence, even when it is lacking, which allows one to maintain their currently held position.

Research on the continued influence effect of misinformation offers insights into how mental models and memory processes contribute to the persistence of misinformation. People construct causal chains of events (van den Broek, 1990), in which misinformation may play a causal role (Johnson and Seifert, 1994). Correction, especially when it does not provide a causal alternative, can disrupt the causal structure supported by misinformation and leave people with an incomplete model, such that people continue to resort to misinformation for comprehension (Johnson and Seifert, 1994). Theories regarding information retrieval offer alternative, complementary explanations. People are susceptible to various reactivation and retrieval failures, such as misattributing the source of the misinformation (vs. corrective information), insufficiently linking a correction to the misinformation in memory such that misinformation is retrieved unchecked despite the correction, and selectively retrieving misinformation in an automatic manner where the false tag attached to it by correction is not co-activated (for a review, see Ecker et al., 2022; Lewandowsky et al., 2012). Furthermore, insofar as misconceptions are formed in memory and that they are often inevitably repeated in a correction, misinformation becomes easier to process with greater familiarity, fluency, and accessibility, which may serve as cues that imply its truth and hedonic value and lead to more positive evaluations (Alter and Oppenheimer, 2009; Swire et al., 2017; Winkielman et al., 2003). Indeed, while corrections may suppress misinformation, they do not replace it (Gordon et al., 2017; Shtulman and Valcarcel, 2012); rather, they coexist and compete such that corrections have to override and inhibit misinformation for beliefs to be updated (Potvin et al., 2015; Trevors, 2019). This process, however, requires effortful, deliberate thinking, which might be hindered by cognitive miserliness or the tendency to default to less costly processing mechanisms (Stanovich, 2021; Pennycook and Rand, 2019). Hence, we have Proposition 5:

**Proposition 5**: The impact and persistence of misinformation are driven by various cognitive fallacies, with or without the motivational forces.

## Persistence of misinformation effects as consequences

Collectively, the motivation and cognitive mechanisms reviewed in the previous section can give rise to various forms of resistance to corrective efforts, which ultimately contribute to the polarization and the persistence of misinformation effects. First, individuals might engage in selective exposure and attention where they selectively access and attend to (mis)information that is consistent with their prior belief and avoid information that contradicts it (Guess et al., 2018; Hart et al., 2009; Knobloch-Westerwick and Meng, 2009), a strategy that is further facilitated by social media environments (Franziska et al., 2019; Spohr, 2017). This means that corrections have a difficult time reaching their target audience in a naturalist setting and, even when it does, individuals tend to place limited cognitive resources on it and are less likely to retain it.

Resistance may also manifest as biased assimilation and weighting. Biased assimilation is the tendency to perceive attitude-congruent information as more valid than attitude-incongruent information (Ahluwalia, 2000; Lord et al., 1979). When corrective information is difficult to refute, individuals resort to relative weighting where they assign less weight to attitude-inconsistent attributes and attach more weight to attitude-consistent ones in their decision-making (Ahluwalia, 2000). They may also reduce the impact of attitude-inconsistent information on their overall judgment of or attitude toward an issue (Ahluwalia, 2000). Put simply, individuals may utilize the information in a distorted way that allows them to sustain their existing beliefs.

Biased perception represents another group of strategies that are frequently employed in response to corrections. It is concerned with cognitive responses to different aspects of a persuasive message, such as its content/arguments, source, and intent (Shen et al., 2009). This can be reflected as, for example, source derogation, which involves questioning the credibility and/or expertise of the source of counterattitudinal information (Cameron et al., 2002; Zhou and Shen, 2022). Similarly, people may develop counterarguments against and refute the content of the attitude-inconsistent message (Taber and Lodge, 2006) as well as challenge the strategy used in the message (Fransen et al., 2015). They may also perceive a stronger manipulative intent from corrective messages (Shen et al., 2009), which is known to trigger reactance (Brehm, 1966), a motivational state marked by anger and critical cognitions that leads to persuasion failure (Dillard and Shen, 2005).

The ultimate manifestation of resistance lies in the boomerang or backfire effect (Hart and Nisbet, 2012; Nyhan and Reifler, 2010), which occurs when a corrective message produces effects opposite to what is intended, resulting in individuals developing an even stronger belief in misinformation. This effect, alongside selective exposure and attention, biased assimilation and weighting, and biased perception, in turn, catalyzes polarization not only on an individual scale where belief in misinformation becomes more entrenched and extreme but also at the societal level such that its dividedness is further exacerbated. Together, these processes illuminate why corrective efforts fail and why the effects of misinformation persist.

## Inoculation as a prebunking strategy

As we have reviewed above, the intricate interplay of various motivational and cognitive mechanisms driving individuals who have a prior belief in misinformation prompts the adoption of diverse strategies to resist correction and preserve their existing view. As such, unsurprisingly, correction strategies such as fact-checking often prove ineffective in countering these misconceptions (Ecker et al., 2014; Seifert, 2014; Thorson, 2016). Meta-analytical evidence documented large effects for the persistence of misinformation despite debunking and observed that a more detailed debunking message was associated with a stronger misinformation-persistence effect (Chan et al., 2017). The challenge is particularly apparent when it comes to real-world misinformation, with research suggesting that the effectiveness of debunking real-world misinformation may diminish by 60% in comparison to constructed misinformation due to Walter and Murphy (2018). Indeed, a recent meta-analysis of correction effects in science-relevant misinformation found a non-significant effect, indicating that efforts to debunk misinformation on issues such as COVID-19 and climate change were overall not successful (Chan and Albarracín, 2023). While corrective messages may be more successful in certain instances, it is evident that they do not eliminate the effect of misinformation (Walter and Tukachinsky, 2020). With that in mind, in this section, we focus our discussion on inoculation as a promising prebunking strategy for mitigating misinformation effects.

### Psychological inoculation theory

The concept of inoculation in persuasion draws from a medical analogy: just as human bodies can be immunized against viruses, our attitudes and beliefs can also be shielded from persuasive attacks (McGuire, 1964). With vaccination, individuals receive a weakened form of the virus, which stimulates the production of antibodies and strengthens the immune system without causing illness itself to safeguard against potential threats from the virus. Similarly, in the persuasion context, a mild version of a counterattitudinal message that can activate defense mechanisms but is not too strong to persuade can confer resistance to counterinfluence.

There are two main parts to an inoculation message: a forewarning message indicating an impending attack on one’s current views and a refutational preemption treatment that provides content that one may employ to refute challenges to their views (Pfau et al., 1997; Compton, 2013). To elaborate, a forewarning message is closely relevant to a perceived threat as one of the key mechanisms in inoculation theory. A prerequisite for inoculation to succeed is for people to develop threat perceptions as they lead to the realization of the vulnerability of their beliefs, which in turn motivates the building of defense systems (Compton and Pfau, 2005; Petty and Cacioppo, 1986). Although the mere presence of counterattitudinal messages can generate threat perceptions (i.e., intrinsic threat, McGuire, 1964), the presence of a forewarning message tends to be more effective (McGuire and Papageorgis, 1962). Refutational preemption, on the contrary, involves a two-sided message that presents a weakened form of arguments from an anticipated attack message along with counterarguments against the arguments. In addition to this more passive refutation approach, individuals are sometimes instructed to develop their own refutation material (Compton and Ivanov, 2013; McGuire and Papageorgis, 1961). Regardless of the approach, refutational preemption serves two main functions: to provide content to be used to refute potential attacks and to allow the practice of counterarguing (Compton and Pfau, 2005; Wyer, 1974). It should be noted that counterarguing, a cognitive process that occurs postinoculation treatment, is not the same and goes beyond refutational materials presented in a refutational preemption treatment (Compton, 2013). It is this cognitive process—motivated by the perceived threat from impending attacks—that confers resistance (Insko, 1967).

Notably, while McGuire and colleagues initially limited the beliefs that could be protected by inoculation to cultural truisms (i.e., widely shared beliefs such as the benefits of tooth brushing where an attack seems impossible, McGuire, 1964), the application of inoculation is no longer confined to non-controversial topics. Indeed, the theory has been shown to successfully confer resistance to attitudes in a wide range of controversial domains such as genetically modified food and vaccines (Banas and Rains, 2010; Compton and Pfau, 2005). In addition, much has been done to address whether the efficacy of inoculation is observed when the attack message presents the same arguments that are countered in the refutational preemption treatment (i.e., “refutational-same”) extends to situations where the attack message raises novel arguments that differ from those addressed in the treatment (i.e., refutational-different). Evidence from decades of research shows that “refutational-same and -different” treatments are equally effective (Banas and Rains, 2010; McGuire, 1962), suggesting that inoculation offers an umbrella or blanket of protection. Further, inoculation may even offer cross-protection such that its protection spillover from one topic to other related topics (e.g., from condom use to binge drinking, Parker et al., 2012, 2016).

### Applying inoculation to prebunk misinformation

Given that inoculation serves as a preemptive measure to counter misinformation before its adoption, it likely provides a more effective solution than corrective measures applied after exposure. Research applying inoculation to mitigate misinformation has generated important insights, supporting its utility. The majority of the early study was done in the context of climate change. For example, van der Linden et al. (2017a), testing the effectiveness of inoculation against the prevailing misinformation that there is no consensus on human-caused climate change, found that the positive impact of a message emphasizing the scientific consensus on the issue was largely preserved by inoculation. Importantly, this effect held for both Democrats and Republicans. In addition, research shows that inoculation produces full protection against climate change misinformation with a one-week delay between the treatment and the attack, demonstrating the longevity of the inoculation effects (Maertens et al., 2020). Beyond climate change, inoculation has been shown to be effective in conferring resistance to misinformation in the realms of health (e.g., Jiang et al., 2022; Geegan et al., 2023), politics (e.g., Zerback et al., 2021), and marketing (e.g., Boman and Schneider, 2021), to name a few.

Building on the umbrella protection effect, scholars have further extended this research by developing a “broad-spectrum immunity” approach to inoculation that is not specific to the claims or the topics of misinformation (Lewandowsky and Van Der Linden, 2021). Cook et al.’s (2017) study represents one of the earliest studies that took this approach. In their study, they developed logic-based refutations that put into question the misleading techniques underlying misinformation. The findings of their study suggested that this strategy was effective in neutralizing the effect of misinformation (Cook et al., 2017). Similarly, Roozenbeek and van der Linden (2019a) found that playing a game that involves creating news articles using various misleading tactics successfully inoculated individuals against fake news. Testing of specific techniques that are commonly used in misinformation, such as impersonation, use of emotional language, polarization, conspiracy theories, trolling, discrediting, *ad hominem* attacks, incoherent arguments, scapegoating, and false dichotomies, showed that inoculation against these techniques, either with an active or passive approach, improved people’s ability to resist misinformation (Roozenbeek et al., 2022; Roozenbeek and van der Linden, 2019b). In sum, there is strong evidence that inoculation that targets manipulation tactics employed in the production of misinformation can effectively generate broad-spectrum immunity to misinformation (see also Basol et al., 2020, 2021; Maertens et al., 2021; Roozenbeek et al., 2020, 2021; Roozenbeek and van der Linden, 2020).

Another important extension of inoculation scholarship focuses on its potential to spread protection to build a societal-level immunity against misinformation, analogous to herd immunity in a medical context. When a large population is inoculated and becomes immune to misinformation, we may effectively limit and control its spread (Lewandowsky and Van Der Linden, 2021; van der Linden et al., 2017b). There is growing evidence that inoculation not only builds resistance but also increases willingness to talk about contested issues (Lin and Pfau, 2007; Lin, 2022). Postinoculation interpersonal conversations, in turn, reinforce the treatment effects and spread the treatment to a larger audience (Ivanov et al., 2012). Indeed, Ivanov et al. (2015) observed that inoculation increased advocacy-driven talk, in which individuals share both treatment-specified and novel arguments, shedding light on its diffusing potential. Much research on the effect of campaign-induced interpersonal communication suggests that the treatment diffused through postinoculation talk can then successfully build resistance among its recipients (Dillard et al., 2022; Jeong and Bae, 2018; Southwell and Yzer, 2007). In sum, inoculation may promote interpersonal talk that spreads the treatment, which in turn may protect the conversation partners from misinformation and ultimately, through this process, help build immunity at a larger scale. It is no surprise that the psychological inoculation strategy has witnessed increased applications (Serhan, 2024). Hence, we have Proposition 6:

**Proposition 6**: Psychological inoculation is an effective strategy to mitigate the impact and persistence of misinformation.

## Discussion

In this article, framing responses to corrective information as a particular case of processing counterattitudinal information, we offer a motivational and cognitive account of the persistence of misinformation. Individuals are often motivated, sometimes automatically so, to preserve their existing attitudes. Even in the absence of motivations, the process of changing and updating beliefs has proven to be challenging due to various cognitive fallacies and mental mechanisms. Together, these motivational and cognitive factors give rise to multiple forms of resistance strategies, which in turn contribute to the entrenchment of misinformation effects. Understanding these underlying processes helps elucidate why debunking efforts frequently fail to be effective. A preemptive strategy, in contrast, has shown promise. By reviewing psychological inoculation theory and its application in prebunking misinformation, we present evidence demonstrating that inoculation against either the claims or manipulative techniques used in misinformation can confer resistance, thereby protecting individuals from misinformation. Further, through interpersonal processes, such inoculation efforts have the potential the spread and establish protection at the societal level.

## Data Availability

The original contributions presented in the study are included in the article/supplementary material, further inquiries can be directed to the corresponding author.
